# Supplementary material for: MuLan-Methyl—multiple transformer-based language models for accurate DNA methylation prediction
Source: Gigascience. 2023 Jul 25;12:giad054. doi: 10.1093/gigascience/giad054 (PMC10367125; doi:10.1093/gigascience/giad054)
Supplement: giad054_Supplemental_Files [file giad054_supplemental_files.zip › MuLan-Methyl_supplementary_Figures.docx]

**Supplementary Tables and Figures**

**Table S1. iDNA-MS dataset statistics.**

**Table S2. The comparison of model performance on the iDNA-MS independent test set between MuLan-Methyl and its sub-models, as well as with the previous studies.**

**
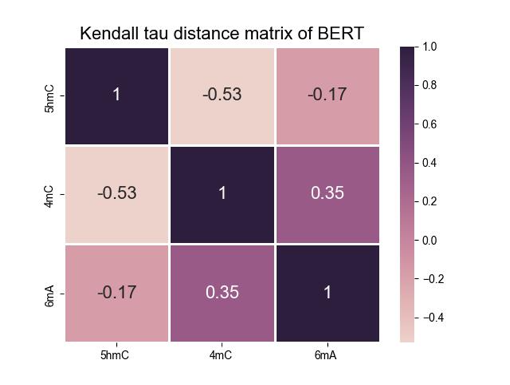

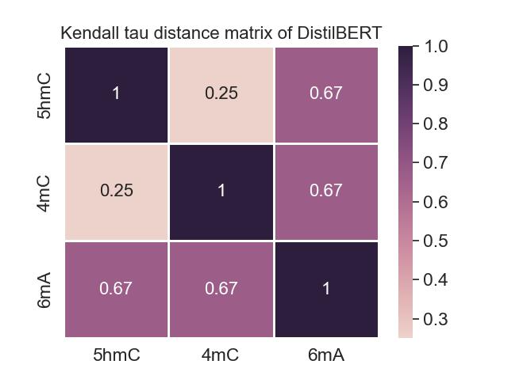

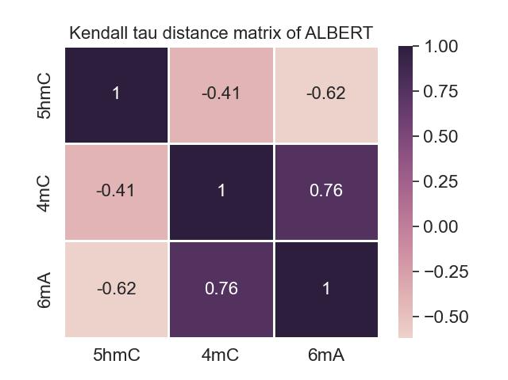

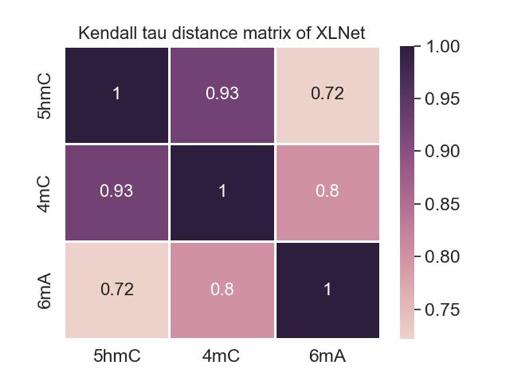

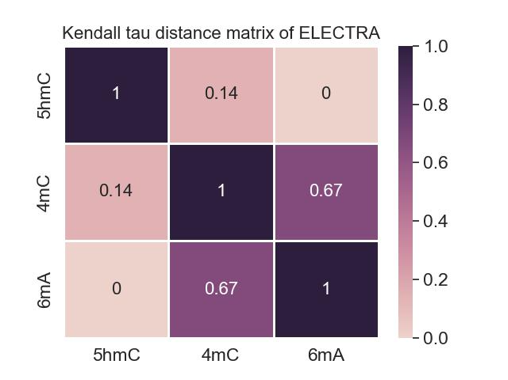
**

**Figure S1.** Heatmap of Kendall tau distance matrix for exploring the ranking correlation, where the rank is obtained by comparing AUC with the other sub-models on each methylation-site type of each sub-model.

**Figure S2.** Boxplot of top 10 tokens with the highest average attention scores for the remaining combinations of methylation types and taxonomic lineage.

**Figure S3.** Heatmap of the average importance score for each position of a 41 bp DNA sequence obtained by merging 6mer fragments for each remaining combination of methylation types and taxonomic lineage.

**Figure S4.** Heatmap of the impact between DNA sequence and its taxonomy lineage for each remaining combination of methylation types and taxonomic lineage.
