# Supplementary material for: MuLan-Methyl—multiple transformer-based language models for accurate DNA methylation prediction
Source: Gigascience. 2023 Jul 25;12:giad054. doi: 10.1093/gigascience/giad054 (PMC10367125; doi:10.1093/gigascience/giad054)
Supplement: giad054_Response_to_Reviewer_Comments_Original_Submission [file giad054_response_to_reviewer_comments_original_submission.pdf]

Author's Response To Reviewer Comments

Close

Reviewer #1:
Zeng et al. proposed an ensemble framework for identifying three type DNA-methylation sites, and performed a benchmark comparison in multiple species' genomic data. This paper give a valuable study on how ensemble transfer learners works and the predictability in different species. My suggestion is the manuscript acceptable with following minor revision:
1. Calculated a consensus ranking using Kendall's tau rank distance method for each method in Figure 2-C.

Response: Kendall tau distance is a distance function for counting the number of pairwise disagreements between two ranking lists, as reviewer’s suggestions, for each transformer-based language model, we calculated the Kendall tau distance of pairwise ranking lists between three methylation types, where each ranking list records this model’s AUC rank over the five models, AUC was obtained by running the model on datasets belonging to this methylation type.
for the model’s AUC ranking among all five models while predicting all test sets for this methylation type. We visualize the results for each transformer-based language model by heatmap and include them in the supplementary file.

2. the multi-head self- attention and self-attention head formula should redescribed by following this paper: https://arxiv.org/pdf/1706.03762.pdf

Response: We revised the formula of multi-head self-attention and the formula of self-attention head according to the paper that the reviewer recommended.

3. MLM and MuLan-Methyl mixed in some cases, which need be used in a consensus way.
Response: Our manuscript uses MLM to represent the abbreviation of Masked Language Modelling, a classic approach to pre-training a language model from scratch, which is a different concept from MuLan-Methyl.

Reviewer #2: In this manuscript, the authors present MuLan-Methyl, a deep-learning framework for predicting 6mA, 4mC, and 5hmC sites. They use DNA sequence and taxonomic identity as features, and implement five popular transformer-based language models in MuLan-Methyl. MuLan-Methyl is open-sourced, and a web server is also provided for users to access it. Overall, I think the methodology of MuLan-Methyl is clear and innovative, and the experiments seem comprehensive. However, I do have several concerns that I believe should be addressed before the paper is accepted by GigaScience.

Major
1. One major concern is that, in my opinion, DNA methylation is dynamic. Cytosines in the same position of the DNA sequence may have different methylation status in different samples, different cells, or even in different development stages of a cell. So, how can we predict the methylation status of a site based on only its sequence (and taxonomic identity)?

Response: Yes, DNA methylation is dynamic, to some degree. Nevertheless, as previous work and our work shows, methylation can be predicted from genomic sequence and taxonomic identity, to some extent. Here our focus is on phrasing DNA methylation prediction task as a natural language processing (NLP) problem, based on sequence and taxonomy.

-- The authors should clarify that in what cases, MuLan-Methyl (as well as other methods that use only DNA sequence) can be used to study DNA methylation, in Introduction or Discussion section.

Response: In the Introduction, we now emphasise that methylation is a dynamic process, yet existing machine-learning techniques are able to predict methylation state from genomic sequence with some degree of accuracy. The performance of existing techniques shows that prediction from sequence is possible and our aim here is to improve such predictions. We have also added a paragraph on this to the Discussion.

-- The authors discuss motifs in Fig. 3, but only for positive samples. How about the motif distribution in the negative samples? Can I understand that this method is actually for discovering motifs (or sequence structures) that are highly correlated with methylation?

Response: Exactly as understood by the reviewer, we study the DNA methylation motifs regarding the positive samples by the self-attention mechanism of the transformer architecture since we are focused on exploring DNA motifs that associate with DNA methylation, which we state in the introduction section and the result section of the revision.

-- How is the performance of MuLan-Methyl without taxonomic identity?

Response: As the reviewer's suggestion, we ran MuLan-Methyl on the processed independent dataset in which we removed the corresponding taxonomic lineage descriptions, after evaluating by AUC evaluation metrics, the AUC of each combination of three methylation-site types and 12 taxonomic lineages was significantly lower than the AUC calculated by running the MuLan-Methyl on the processed independent test set with the corresponding taxonomic lineages.
The reason could be that MuLan-Methyl is trained on the dataset with the sample's taxonomic identity, and the visualisation from MuLan-Methyl interpretability (Figure 3G-I) demonstrates the correlations between the taxonomic lineages and the methylation sites (center of the sample) of positive samples, showing the necessity of a sample's taxonomy identity.
Fine-tuning MuLan-Methyl on the training dataset also without taxonomic identity information would have a better performance for predicting on the test dataset without taxonomic identity.

2. The authors compared MuLan-Methyl against iDNA-ABF and iDNA-ABT, especially on the independent test set (Fig. 2E). I think the authors should clarify that whether they trained the models of the three methods using the same training datasets. If not, the authors should clarify the reason.

Response: MuLan-Methyl and the other two frameworks are essentially trained on the same benchmark dataset provided by the iDNA-MS study, iDNA-ABF and iDNA-ABT both contain 17 classifiers, and each is trained on the sub-datasets generated by splitting iDNA-MS according to the methylation types and taxonomic lineages. Meanwhile, MuLan-Methyl gives rise to 3 classifiers, each trained on the sub-dataset generated by splitting the iDNA-MS according to methylation types.
We clarified it in the result section as the reviewer suggests.

3. I'm curious about the computational efficiency of MuLan-Methyl. How many parameters in its model? Does MuLan-Methyl have advantages over other methods in terms of computational efficiency?

Response: MuLan-Methyl consists of five transformer-based language models, we listed each transformer-based language model's parameter number as below.

Model Number of parameters
MuLan-Methyl-BERT 105,242,882
MuLan-Methyl-DistilBERT 62,714,114
MuLan-Methyl-ALBERT 11,045,122
MuLan-Methyl-XLNet 111,934,466
MuLan-Methyl-ELECTRA 29,336,578

In terms of the computational efficiency of MuLan-Methyl compared to the other two methods, MuLan-Methyl proposed three classifiers for methylation-site types, whereas the other methods built a separate

classifier for each taxonomic lineage and each methylation-site type, resulting in 17 classifiers. The number of models that MuLan-Methyl need to train and test on is less than the others, thus it has better computational efficiency than other models to some extent.

Minor
1. I don't understand why the references were not ordered from 1 in the main text.

Response: We apologize for the disordering of references in the manuscript. We corrected it in the revision.

2. I suggest that the authors re-organize the Introduction section. There are too many small paragraphs in this section.

Response: We re-organized the introduction section in the revision as the reviewer suggests.

3. At the end of Page 2, "The type 4mC type is present in 4 species" should be corrected.

Response: We apologize for the mistake, we corrected it to “The 4mC methylation type is present in 4 species” in the revision.

Close
