# Supplementary material for: MuLan-Methyl—multiple transformer-based language models for accurate DNA methylation prediction
Source: Gigascience. 2023 Jul 25;12:giad054. doi: 10.1093/gigascience/giad054 (PMC10367125; doi:10.1093/gigascience/giad054)
Supplement: giad054_Response_to_Reviewer_Comments_Revision_1 [file giad054_response_to_reviewer_comments_revision_1.pdf]

Author's Response To Reviewer Comments

Close

In addition, please register any new software application in the bio.tools and SciCrunch.org databases to receive RRID (Research Resource Identification Initiative ID) and biotoolsID identifiers, and include these in your manuscript. Computational workflows should be registered in workflowhub.eu and the DOIs cited in the relevant places in the manuscript. These will facilitate tracking, reproducibility and re-use of your tool.

Response: We have registered the approach with bio.tools and have obtained an RRID, both are now listed under Data Availability.

Reviewer #2: The authors have addressed most of my concerns. However, I still have one minor concern about the computational efficiency. The response of the authors is not convincing by only saying "The number of models that MuLan-Methyl need to train and test on is less than the others, thus it has better computational efficiency than other models to some extent". If possible, I strongly suggest that the authors show some data to compare how much time and resources (GPU/CPU/RAM) needed by each method.

Response: a detailed comparison is difficult to perform because we were not able to replicate one of the approaches (iDNA-ABT) on our own server. However, we were able to perform a cursory comparison of run time and have added the following sentence to the manuscript (at the end of the section "Comparison with existing methods"):

"A cursory comparison suggests that MuLan-Methyl and iDNA-ABF have similar reported run times (albeit using different GPUs), whereas iDNA-ABT runs about ten times faster."

Close
