# Supplementary material for: MuLan-Methyl—multiple transformer-based language models for accurate DNA methylation prediction
Source: Gigascience. 2023 Jul 25;12:giad054. doi: 10.1093/gigascience/giad054 (PMC10367125; doi:10.1093/gigascience/giad054)
Supplement: giad054_Response_to_Reviewer_Comments_Revision_4 [file giad054_response_to_reviewer_comments_revision_4.pdf]

Author's Response To Reviewer Comments

Close

Here are the updated files

Close
