# Supplementary material for: MuLan-Methyl—multiple transformer-based language models for accurate DNA methylation prediction
Source: Gigascience. 2023 Jul 25;12:giad054. doi: 10.1093/gigascience/giad054 (PMC10367125; doi:10.1093/gigascience/giad054)
Supplement: giad054_Reviewer_2_Report_Original_Submission — Jianxin Wang -- 4/19/2023 Reviewed [file giad054_reviewer_2_report_original_submission.pdf]

Reviewer Report

Title: MuLan-Methyl - Multiple Transformer-based Language Models for Accurate DNA Methylation Prediction

Version: Original Submission **Date:** 4/19/2023

Reviewer name: Jianxin Wang

Reviewer Comments to Author:

In this manuscript, the authors present MuLan-Methyl, a deep-learning framework for predicting 6mA, 4mC, and 5hmC sites. They use DNA sequence and taxonomic identity as features, and implement five popular transformer-based language models in MuLan-Methyl. MuLan-Methyl is open-sourced, and a web server is also provided for users to access it. Overall, I think the methodology of MuLan-Methyl is clear and innovative, and the experiments seem comprehensive. However, I do have several concerns that I believe should be addressed before the paper is accepted by GigaScience.
Major
1. One major concern is that, in my opinion, DNA methylation is dynamic. Cytosines in the same position of the DNA sequence may have different methylation status in different samples, different cells, or even in different development stages of a cell. So, how can we predict the methylation status of a site based on only its sequence (and taxonomic identity)?
-- The authors should clarify that in what cases, MuLan-Methyl (as well as other methods that use only DNA sequence) can be used to study DNA methylation, in Introduction or Discussion section.
-- The authors discuss motifs in Fig. 3, but only for positive samples. How about the motif distribution in the negative samples? Can I understand that this method is actually for discovering motifs (or sequence structures) that are highly correlated with methylation?
-- How is the performance of MuLan-Methyl without taxonomic identity?
2. The authors compared MuLan-Methyl against iDNA-ABF and iDNA-ABT, especially on the independent test set (Fig. 2E). I think the authors should clarify that whether they trained the models of the three methods using the same training datasets. If not, the authors should clarify the reason.
3. I'm curious about the computational efficiency of MuLan-Methyl. How many parameters in its model? Does MuLan-Methyl have advantages over other methods in terms of computational efficiency?
Minor
1. I don't understand why the references were not ordered from 1 in the main text.
2. I suggest that the authors re-organize the Introduction section. There are too many small paragraphs in this section.
3. At the end of Page 2, "The type 4mC type is present in 4 species" should be corrected.

Methods

Are the methods appropriate to the aims of the study, are they well described, and are necessary controls included? Choose an item.

Conclusions

Are the conclusions adequately supported by the data shown? Choose an item.

Reporting Standards

Does the manuscript adhere to the journal's guidelines on minimum standards of reporting? Choose an item.

Choose an item.

Statistics

Are you able to assess all statistics in the manuscript, including the appropriateness of statistical tests used? Choose an item.

Quality of Written English

Please indicate the quality of language in the manuscript: Choose an item.

Declaration of Competing Interests

Please complete a declaration of competing interests, considering the following questions:

- Have you in the past five years received reimbursements, fees, funding, or salary from an organisation that may in any way gain or lose financially from the publication of this manuscript, either now or in the future?
- Do you hold any stocks or shares in an organisation that may in any way gain or lose financially from the publication of this manuscript, either now or in the future?
- Do you hold or are you currently applying for any patents relating to the content of the manuscript?
- Have you received reimbursements, fees, funding, or salary from an organization that holds or has applied for patents relating to the content of the manuscript?
- Do you have any other financial competing interests?
- Do you have any non-financial competing interests in relation to this paper?

If you can answer no to all of the above, write 'I declare that I have no competing interests' below. If your reply is yes to any, please give details below.

I declare that I have no competing interests

I agree to the open peer review policy of the journal. I understand that my name will be included on my report to the authors and, if the manuscript is accepted for publication, my named report including any attachments I upload will be posted on the website along with the authors' responses. I agree for my

report to be made available under an Open Access Creative Commons CC-BY license (http://creativecommons.org/licenses/by/4.0/). I understand that any comments which I do not wish to be included in my named report can be included as confidential comments to the editors, which will not be published.

Choose an item.

To further support our reviewers, we have joined with Publons, where you can gain additional credit to further highlight your hard work (see: https://publons.com/journal/530/gigascience). On publication of this paper, your review will be automatically added to Publons, you can then choose whether or not to claim your Publons credit. I understand this statement.

Yes Choose an item.
